# Implementation of Chemical Health, Safety, and Environmental Risk Assessment in Laboratories: A Case-Series Study

**DOI:** 10.3389/fpubh.2022.898826

**Published:** 2022-06-14

**Authors:** Farin Fatemi, Alireza Dehdashti, Mohammadreza Jannati

**Affiliations:** ^1^Department of Occupational Health, Research Center of Health Sciences and Technologies, Semnan University of Medical Sciences, Semnan, Iran; ^2^Social Determinants of Health Research Center, Semnan University of Medical Sciences, Semnan, Iran; ^3^Student Research Committee, Semnan University of Medical Sciences, Semnan, Iran; ^4^Department of Occupational Health and Safety, Memorial University of Newfoundland, St. John's, NL, Canada

**Keywords:** chemicals, risk assessment, academic, laboratories, safety

## Abstract

**Introduction:**

Characterizing risks associated with laboratory activities in universities may improve health, safety, and environmental management and reduce work-related diseases and accidents. This study aimed to develop and implement a chemical risk assessment method to determine and prioritize more hazardous chemicals in the academic laboratories.

**Methods:**

A case-series study was conducted at five academic laboratories and research facilities of an Iranian medical sciences university in 2021. A risk assessment was developed and implemented in three phases to identify, evaluate, and classify potential risks and hazards. The approach provided an innovative tool for evaluating and prioritizing risks in chemical laboratories. Hazards were classified on a five-level scale. The technique reviewed both quantitative and qualitative data and pieces of evidence using Laboratory Safety Guidance (OSHA), Occupational Hazard Datasheet (ILO), the standards of the American Conference of Governmental Industrial Hygienists (ACGIH), International Agency for Research on Cancer (IARC), and National Fire Protection Agency (NFPA) codes.

**Results:**

Overall, the frequency of risks rated from “moderate” to “very high” levels was determined for the health hazards (9.3%), environmental hazards (35.2%), and safety hazards (20.4%). Hydrochloric acid had a high consumption rate in laboratory operations and received the highest risk levels in terms of potential hazards to employees' health and the environment. Nitric acid, Sulfuric acid, Formaldehyde, and Sodium hydroxide were assessed as potential health hazards. Moreover, Ethanol and Sulfuric acid were recognized as safety hazards. We observed adequate security provisions and procedures in academic laboratory operations. However, the lack of awareness concerning health, safety, environmental chemical hazards, and inappropriate sewage disposal systems contributed to the increasing levels of laboratory risk.

**Conclusions:**

Chemicals used in laboratory activities generate workplace and environmental hazards that must be assessed, managed, and risk mitigated. Developing a method of rating health, safety, and environmental risks related to laboratory chemicals may assist in defining and understanding potential hazards. Our assessment suggested the need for improving the risk perception of individuals involved in handling chemicals to prevent exposure from workplace duties and environmental pollution hazards.

## Introduction

Laboratories and research facilities are considered a fundamental part of universities playing a crucial role in preparing students and researchers to obtain skills that are valuable in their future careers (1). The presence of numerous chemicals in laboratories has faced safety and health managers with challenges in estimating their risks and hazards. The chemicals and equipment that are used by laboratory personnel and students present a number of serious, sometimes life-threatening hazards and accidents. Laboratory managers are responsible to protect their personnel and students from exposure to chemical, biological, and physical hazards (2). Therefore, the presented risk assessment method for the academic laboratories and applying prevention and mitigation measures in this study enable the laboratory managers to do their responsibility to their personnel and students.

A survey by OSHA has reported that the potential hazards associated with conducting research at laboratories in academic institutions were 11 times more dangerous as compared to commercial laboratories in a range of industrial sectors with labs (3). Literature review on the safety and health of laboratories in higher education institutions has shown many laboratory incidents leading to fatalities and injuries caused by fires, explosions, and equipment resulting in debilitating injuries and death (4). Previous studies on health-related hazards have reported both acute and chronic poisonings following exposure to various chemicals in laboratory environments (5). Moreover, laboratory wastewater consists of hazardous chemicals that have been considered a substantial environmental threat (6). In the United States, about 18% of occupational accidents in higher education institutions were related to laboratory environments and in approximately one-third of accidents, students were the main victims (7–9). A review of reported cases in the literature evidence suggests that the trend of accidents was on the rise in academic laboratories over the past several years (10, 11). Lack of awareness of various safety and health hazards has triggered accidents, mainly related to the unsafe work practices of chemicals and equipment in laboratories (12).

Integrated health, safety, and environmental risk assessment would be beneficial in understanding risks, evaluating hazards, and planning a strategy to prevent accidents in laboratories (13, 14). International occupational safety and health organizations have developed standards and instructions to prevent and control hazards in laboratory environments. Training of students and laboratory workers provided a culture of safety, health, and environmental consciousness in dealing with laboratory risks and hazards (15). Although risk assessment has shown to be an efficient approach to identify and introduce appropriate measures to manage risks and hazards, workplace risk levels may differ based on tasks and unsafe acts even in the same work environment. In essence, the laboratory risk assessment should be implemented for individual specific laboratory settings and each work task and role to effectively apply controls (16). Obtaining objective and comprehensive data concerning risks and hazards has presented challenges for health, safety, and risk management professionals in chemical laboratories. Planning a risk assessment requires the definition of an assessing project with an educated team. Hazard prediction and recognition are the beginning or first step to measure the strength of the impact of a threat (2).

Many research activities are performed in chemical laboratories at universities, which are seldom assessed by occupational safety and health engineers (11). This study performed an integrated health, safety, and environmental risk assessment to determine the level of risks for potential workplace exposure in terms of different jobs and work duties in academic lab settings. The process includes prediction, recognition, classification, and evaluation of risks and hazards in chemical laboratories. The plan for adequate measures to prevent and mitigate risks and fitness of work to laboratory personnel and the student will be discussed.

## Methods

### Design and Setting of the Study

A cross-sectional design and action research were applied to develop and conduct a comprehensive risk assessment to determine a range of health, safety, and environmental risks associated with the activities in academic laboratories. This study was implemented at five medical and health sciences laboratories affiliated to Semnan University in 2021.

### Suggested Steps of Risk Assessment

**Figure 1:**
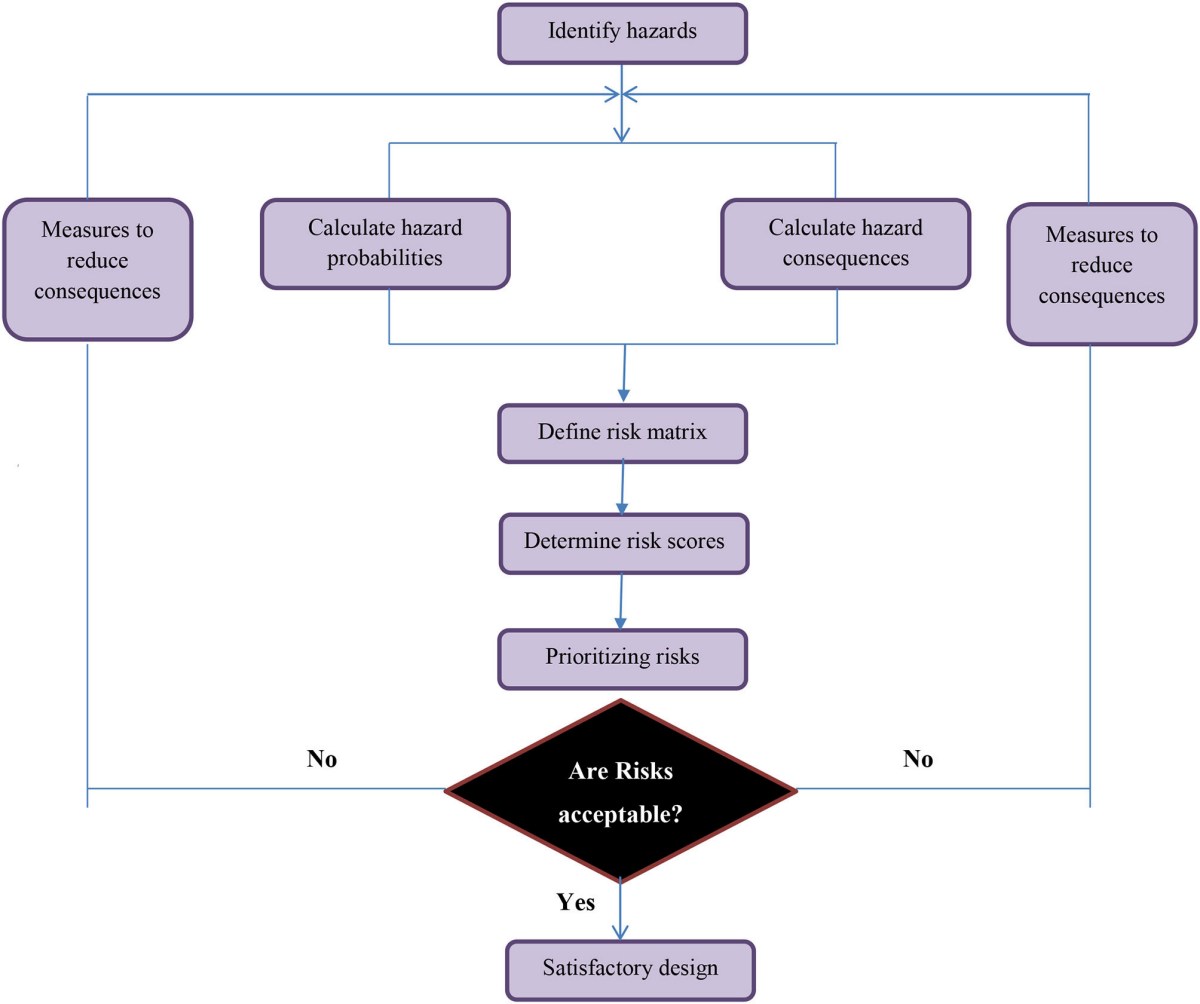
Flowchart of the suggested methodology for risk assessment.

Figure 1 demonstrates the methodology steps proposed for assessing risks in chemical laboratories in university environments. These include developing an integrated risk approach, collecting information to categorize risk factors, calculating risk levels, and proposing health, safety, and environmental measures.

### Development of an Integrated Risk Assessment Approach

Our methodology is based on the use of a structured checklist to integrate the process of predicting and recognizing hazards, evaluating the risks posed by hazards, and managing the risks of hazards in the context of the university laboratory. This technique reviewed both quantitative and qualitative data regarding chemicals, environment, and activities associated with the specific processes, and judgments were confined to a particular laboratory process in isolation.

Recognition of potential risks and hazards in laboratory environments and activities was based on checklists, walk-through observation, and interviews with working individuals in laboratories. We developed a combined behavior-based and process-based checklists to conduct a broader risk assessment for identifying the risk level of work practices and mitigating the associated risks. The study tool was adopted from Laboratory Safety Guidance (OSHA), Occupational Hazard Datasheet (ILO), and the Princeton University Laboratory Safety Manual. The tool consisted of 131 items, which were used to assess working areas, emergency planning, required information and documentation, personal protective equipment, electrical hazards, chemical storage and use, flammable liquids, compressed gases, disposal of chemicals used in the lab, ventilation requirements, security, and training.

### Collecting Information to Categorize Risk Factors

We identified and grouped chemical exposure and hazards according to their properties, work procedures, and occupational potential exposure scenarios by using frequency and work behavior in the laboratories studied.

### Calculating Risk Levels

The laboratory hazard risk rating of a chemical was estimated by multiplying the severity of consequence value by the likelihood of incidence value. For this step, we assembled literature on hazard properties for each chemical from reliable resources to obtain a review of a clear understanding of the safety and health controls. The pieces of literature were reviewed for exposure limits and carcinogenicity of chemical substances as identified by the standards of American Conference of Governmental Industrial Hygienists (ACGIH), Immediately Dangerous to Life or Health Concentrations (IDLH) of toxic substances, and National Fire Protection Agency (NFPA) codes (17, 18).

We used an assessment matrix to conduct a comparative analysis concerning “the severity of consequence” and “the probability of incidence” to determine the risk rating for individual health, safety, and environmental hazards. Our estimates of hazard risk ratings were used to categorize risk into varying levels of risk by applying standard linear scaling. Table 1 demonstrates the matrix of risk levels and expectations of responses required to improve safety and health in the laboratory (ISO 31000) (19).

**Table 1:** Establishing a laboratory hazard and process matrix-based risk system with standard linear scaling (values 1–5) to determine the risk score.

**Likelihood severity**	**1**	**2**	**3**	**4**	**5**
1	1	2	3	4	5
2	2	4	6	8	10
3	3	6	9	12	15
4	4	8	12	16	20
5	5	10	15	20	25
					
**Interpretation**
Very low	1 - ≤ 5	Risk is acceptable and control measures is not necessary			
Low	5.01 - ≤ 10	Risk is low and further studies needed in the future			
Moderate	10.01 - ≤ 15	Risk is intermediate and control measures have to be done in the future			
High	15.01 - ≤ 20	Risk is high and control measures have to be done as soon as possible			
Very high	20.01 - ≤ 25	Risk is very high and control measures have to be done immediately			

### Proposing Health, Safety, and Environmental Measures

The prevention and mitigation of health, safety, and environmental risk measures were proposed based on calculated risk scores.

## Results

In this study, we used a checklist to recognize potential risks and hazards in the laboratory settings. Health, safety, and environmental hazards associated with common chemical laboratory activities and workflow and the percentage of compliance and non-compliance with laboratory guidelines are shown in Table 2.

**Table 2:** Results of hazard analysis checklist based on work processes and behaviors evaluated in university chemical laboratories and verified frequency of compliance and non-compliance with health, safety, and environmental guidelines.

**Laboratory environment and facilities**	**Compliance** **(%)**	**Non-compliance** **(%)**
1. General work environment	59	41
2. Emergency planning	42	58
3. Required information and documentation	20	80
4. Personal protective equipment	25	75
5. Electrical hazards	56	44
6. Chemical storage	56	44
7. Flammable liquids	83	17
8. Compressed gases	87.5	12.5
9. Disposal system	NO^*^	100
10. Ventilation	83	17
11. Security	100	NO^*^
12. Training	17	83
13. Awareness	36	64

* *Not Observed*.

**Figure 2:**
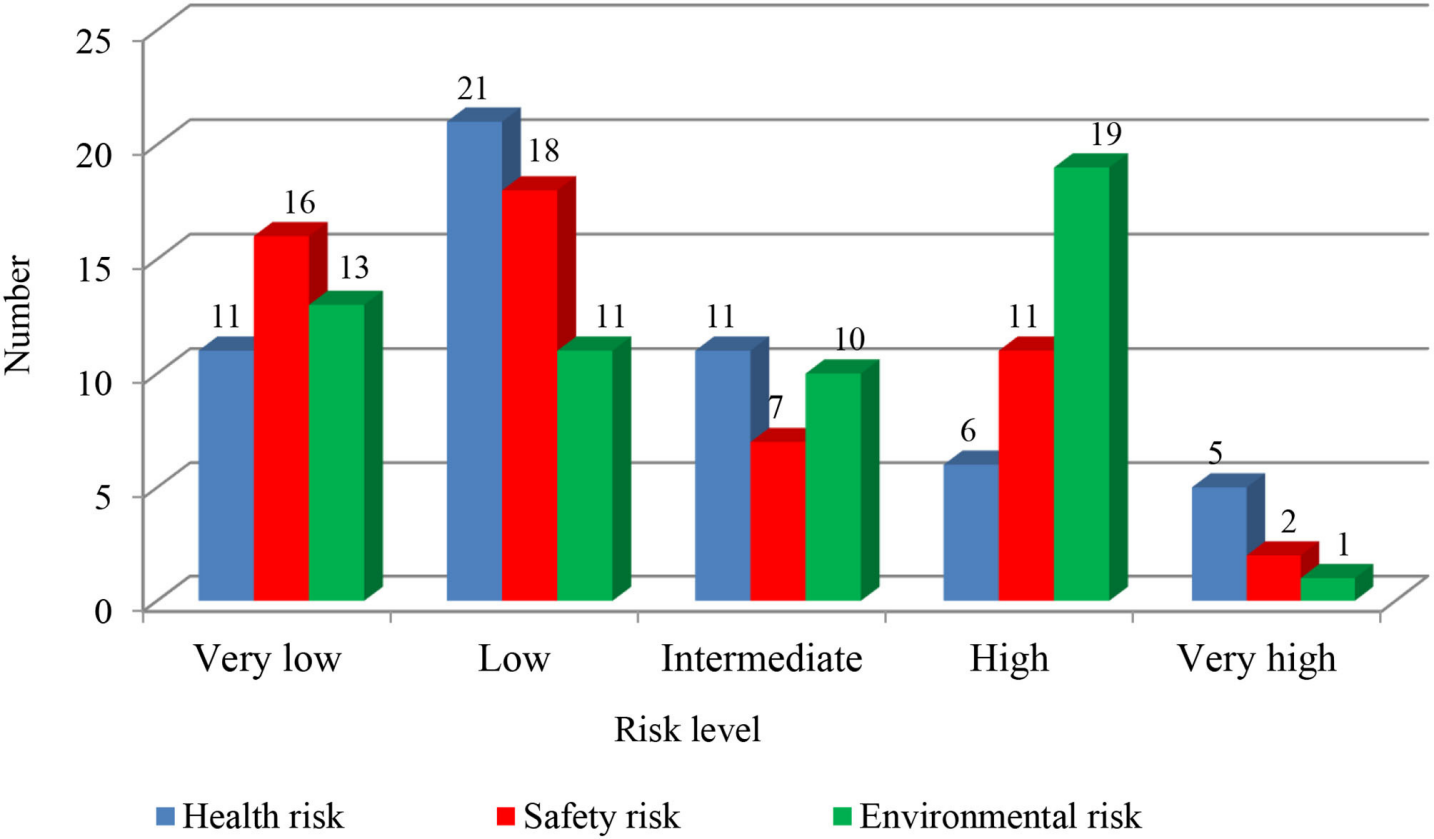
Frequency of chemicals at estimated risk level classes in university laboratory activities.

Our survey of laboratory activities showed that work with compressed gases and flammable liquids was in acceptable compliance with security considerations and safe work procedures. However, the above half of non-compliance was related to the preparation in emergency response situations, not using personal protective equipment, poor inappropriate chemical disposal, treatment of waste products, and awareness and training. The lack of written emergency action plans, chemical hygiene lab procedures, and Safety Data Sheet (SDS) were identified to contribute to operational risks in chemical laboratory activities. The unsafe acts by the lab staff related to waste effluent disposal management mainly included risk factors of improper disposal containment and methods for experiment waste. We observed a lack of compliance in emergency response plans that are mainly associated with inadequate knowledge of staff and students about how to identify the location of fire extinguishers, how to request emergency assistance, and how to communicate potential leak, fire, and explosion scenarios. The unsafe conditions, such as aging electrical cords and plugs and contact with incorrectly grounded devices, were identified to increase operational risks of instruments in laboratories. Additionally, obstructed fire alarm pull stations or inappropriate layout of fire extinguishers in the lab environments increases the reaction time in the occurrence of accidents. Almost all individuals involved in handling chemicals in the laboratories reported they had not received the proper chemical safety training. Our onsite observations showed the unsafe storage of chemicals, which may lead to leakage and increase the possibility of exposure and accidents or high potential for injuries and damages. Students and laboratory workers were more likely not to choose the safe course of action concerning the use of personal protective equipment. For example, a common unsafe act was working in university labs without wearing face and eye or respiratory protection. The absence of proper Protective Personal Equipment (PPE) leads to unsafe exposure and subsequent injury. Furthermore, in chemical laboratories, the users frequently violated safe work procedures during transporting or setting up the experiment or apparatus. We identified many facilities and experiments in compliance with environment, health, and safety codes for handling flammable liquids and compressed gases in chemical laboratories. However, any deviation from the intended experimental steps in laboratory operations could result in severe consequences. The survey evaluated comprehensive health, safety, and environmental hazards of 54 chemicals used in chemical laboratories (Figure 2). The proposed class-based risk assessment involves five levels of classes. The fourth- and fifth-level classes characterize the main risk factors.

A total of 44 risk factors were predicted and recognized as the “high” or “very high” level assessment classes. Potential health hazards recognized at the “very high” level were more frequent when compared to safety and environmental hazards, respectively, accounting for 9.2, 3.7, and 1.8% of the total number of hazards at the “very high” level class. Moreover, the chemicals with the level of “high” risk contributed to a greater number of environmental hazards (35.2%) followed by safety hazards (20.4%) and health hazards (11.1%). The identified health, safety, and environmental hazards of chemicals at the intermediate level were, respectively, 20.4, 13, and 18.5% of the total number of third-level categories, implying that prevention and control actions are required to manage the risks. Additionally, the mean value of 29.7% of the assessed chemicals had very low and low health risk levels. These mean values for safety and environmental hazards were 31.5 and 22.3%, respectively.

Overall, using chemicals in laboratory operations produced a wide range of risk levels. Cyclohexane, Nitric acid, Sulfuric acid, Formaldehyde, and Sodium Hydroxide were classified as “very high” risk levels with a score estimated at 25, accounting for 9.3% of potential hazards to health. Many chemicals (35.2%) were classified at the “high” risk levels involved in environmental hazards. In contrast, few chemicals (1.8%) presented a “very high” risk level to the environment. Table 3 demonstrates the potential health, safety, and environmental hazards of the studied chemicals and the relevant calculated risk scores.

**Table 3:** Health, safety, and environmental risk assessment matrix of common chemicals used in university laboratories.

**Chemicals name**	**Environmental risk**			**Safety risk**			**Health risk**		
	**Probability**	**Severity**	**Risk score**	**Probability**	**Severity**	**Risk score**	**Probability**	**Severity**	**Risk score**
Acetone	4	1	4	4	5	20	4	3	12
Acetic acid	4	4	16	3	4	12	4	3	12
Ethanol	5	3	15	5	5	25	5	3	15
Ammoniac	4	5	20	3	5	15	4	4	16
Benzene	3	3	9	4	4	16	3	5	15
Butanol	4	4	8	4	5	20	2	2	4
Chloroform	4	5	20	4	5	20	2	3	6
Cyclo hexanol	3	2	6	3	5	15	3	2	6
Hydrochloric acid	5	5	25	5	4	20	5	5	25
Hydrogen peroxide	4	4	16	4	3	12	4	4	16
Methanol	3	5	15	5	4	20	3	2	6
Nitric acid	5	4	20	4	4	16	5	5	25
Sulfuric acid	5	4	20	5	5	25	5	5	25
Di chloromethane	4	1	4	4	2	8	3	3	9
Di ethyl ether	3	3	9	4	5	20	3	2	6
Ethylene glycol	2	2	4	2	2	4	2	3	6
Formaldehyde	4	5	20	3	4	12	5	5	25
Isopropanol	3	3	9	4	4	16	3	2	6
Orto toluidine	1	5	5	2	4	8	1	4	4
Toluene	3	3	9	4	4	20	3	3	9
Carbon disulfide	4	3	12	4	5	20	3	4	12
Paraffin	4	1	4	1	2	2	2	2	4
Aluminum sulfate	4	4	16	1	3	3	3	3	9
Arsenic oxide	2	5	10	3	3	9	2	5	10
Barium chloride	2	5	10	1	2	2	2	3	6
Cadmium chloride	3	5	15	1	2	2	3	5	15
Iodine	4	5	20	2	2	4	5	4	20
Ferric sulfate	3	4	12	1	3	3	3	3	9
Ferric chloride	3	3	9	2	1	2	3	4	12
Ammonium carbonate	2	5	10	2	4	8	2	3	6
Ammonium chloride	2	2	4	2	1	2	2	3	6
Asbestos	4	4	8	2	4	8	1	5	5
Brome	2	5	10	3	3	9	2	4	8
Calcium carbonate	3	1	3	1	1	1	3	3	9
Calcium hydroxide	3	4	12	1	3	3	3	4	12
Magnesium oxide	2	5	10	1	2	2	2	3	6
Phenol	4	5	20	2	3	6	2	5	10
Manganese sulfate	4	5	20	1	2	2	2	4	8
Potassium hydroxide	5	4	20	3	3	9	5	4	20
Silver nitrate	3	5	15	2	3	6	3	4	12
Sodium azide	1	5	5	3	2	6	1	4	4
Sodium fluoride	3	5	15	2	2	4	3	4	12
Sodium hydroxide	5	4	20	3	3	9	5	5	25
Mercury	4	5	20	2	3	6	2	4	8
Potassium cyanide	4	5	20	3	4	12	2	4	8
Sodium cyanide	1	5	5	2	3	6	1	4	4
Potassium chromate	4	5	20	2	3	6	5	4	20
Tin chloride	4	5	20	2	3	6	4	4	16
Citric acid	2	2	4	1	2	2	2	2	4
Cobalt chloride	4	5	20	2	3	6	2	4	8
Lead acetate	1	5	5	2	2	4	1	3	3
Lead nitrate	1	5	5	2	4	8	1	4	4
Mercury chloride	4	5	20	3	5	15	1	5	5
Nitrate nickle	1	5	5	2	4	8	1	3	3

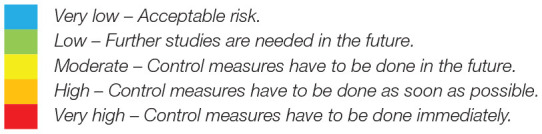

Our risk assessment showed that 25.9% of the laboratory chemicals might be associated with heavy potential exposure as scored at 5 or 4. Moreover, more than half of the laboratory chemicals (25.9%) contributed to the high level of severity outcomes. The results demonstrated that Ethanol and Sulfuric acid presented a “very high” risk level (scored at 25) in safety risk assessment. Furthermore, 27.8 and 44.4% of chemicals were rated high scores of probability and severity, respectively, in the safety risk assessment. Hydrochloric acid was the only chemical that was ranked at the “very high” level in the environmental risk assessment, with a score estimated at 25.

## Discussions

This study assessed health, safety, and environmental risks in academic laboratories that use chemicals for educational and research activities. The variability of chemical use in academic laboratories might lead to various health, safety, and environmental risk factors. Our findings agree with prior research that suggested that educational and research laboratories of academic institutions need to assess their vulnerabilities and plan their own risk mitigation accordingly (20).

Our risk assessment indicated that the percentage of health hazards at the “very high” risk level was higher when compared to the safety and environmental hazards. Overall, the mean values of 13.6, 12.4, and 18.5% of the assessed chemicals were classified in “moderate” to “very high” categories of health, safety, and environmental hazards, respectively. Therefore, health and safety rules must be considered strictly as a priority by the people who work with chemicals in laboratories for reducing the risk of chemical-related diseases and accidents (21). In this study, the laboratory health and safety checklist showed that most non-compliance was linked to the chemical storage and training/awareness sections. The main faults in chemical storage were related to the labeling of cabinets to indicate chemical class and the labeling of chemical containers, particularly when chemicals are transferred from their original containers. Additionally, quantities of chemicals in storage were inconsistent with short-term needs of the assessed laboratories. All of these non-compliances in chemical storage may result in extensive fire or explosion in the laboratories of academic settings. Omidvari et al. found similar results in their study at Azad University in Iran, which reported fire risk and accidents in educational buildings, particularly in laboratories (22).

Due to the importance of training and awareness in reducing exposures, accidents, and injuries, all laboratory workers, such as faculty, staff, and students, should receive laboratory standard training. The training programs should involve chemical safety programs, chemical emergency action plans, and laboratory security plans. After holding the training courses, it should be ensured that the laboratory workers know who and when to use personal protective equipment, how to use emergency equipment, such as eyewashes and safety showers, where SDSs are kept, spill control procedures, emergency procedures, and chemical waste procedures. The previous studies recommended the periodic training courses for laboratory staff and approved the laboratory safety and security curriculum in most faculties in order to increase awareness, safety, and security culture among laboratory workers and allow them to distinguish what to do before, during, and after emergencies (9, 23–25).

Moreover, the general work environment, emergency planning, and required information for chemical laboratories were the other parts of the checklist that involved the highest numbers of non-compliance in this study. Not only allocating one room of the chemistry laboratory to a chemical warehouse has been increased the safety risk but also the layout of chemicals was not in accordance with safety principles and standards for practice. For instance, the chemical storage was not at “least 18 below the sprinkler head or at least 24” below the ceiling. In at least 2 laboratories, not considering the 5S principles for work environment and storage of materials, such as paper goods, plastic containers, boxes, and empty containers, that would fuel to the burning fire was major non-compliance violation. Additionally, the alternative exits, chemicals material safety data sheet (MSDS), safety instructions, Self-Contained Breathing Apparatus (SCBA), and required special security systems or controls to limit access were not available in the assessed laboratories. The lack of an emergency action plan was the other major fault in this study. The findings of this study and similar research studies provide useful information to plan and develop an emergency action plan for the prevention and mitigation of the emergencies and their harmful consequences in the laboratories of academic institutions (26–28). The prevention and mitigation measures should be prioritized for implementation in accordance with available funds and other resources. Prior studies reported low-cost interventions that might involve reducing major risks and their consequences. Planning a safe layout for gas cylinders or fire extinguishers, providing the SDS for all chemicals used in laboratories, using chemical labeling of cabinets and containers, and non-structural mitigation measures are recommended (29, 30).

In the domain of environmental risk assessment, 44.5% of chemicals were classified in “very low” and “low' risk levels, but 55.5% of them were ranked “intermediate” to “very high” risk degrees. The most important chemical environment-related hazard was waste disposal. The lack of an individual sewage system for laboratories and releasing chemicals into the urban sewage system can contaminate the underground water with hazardous chemicals. Previous studies assessed a high level of environmental risk in underground water reservoirs related to hazardous chemical effluents from academic laboratories (31, 32).

## Conclusions

This chemical health, safety, and environmental risk assessment was developed and conducted according to the standards and guidelines set by the international occupational health and safety organizations. The applied approach revealed the significant risks associated with chemicals used at the university laboratories. The instrument developed for this study will be put into good use in helping health and safety engineers to identify and classify potential risks of laboratory operations to health, safety, and environment. Prevention and mitigation measures should be based on detailed risk assessment methods to minimize identified hazards and provide a safe environment to reduce and/or eliminate the occurrence of diseases and injury in laboratories.

Universities should provide training courses in the curriculum on health and safety in laboratories, particularly for new students at the first of each semester, and periodic similar training courses for faculty and staff plays a key role in increasing awareness and risk perception for considering significant risks at the laboratories. Furthermore, inspecting and assessing the laboratories and research facilities by standard laboratory checklists routinely and removing the non-compliance operations at the earliest time are essential in providing a safe work environment.

## Data Availability Statement

The raw data supporting the conclusions of this article will be made available by the corresponding author, without undue reservation.

## Ethics Statement

This study was approved by the Ethics Committee Review Board at Semnan university of Medical Sciences (IR.SEMUMS.REC.1398.131). All the participants signed a consent form and were informed on the purpose of the study prior to interview as per local protocol on research ethics.

## Author Contributions

AD: material preparation, conceptualization, methodology, investigation, writing—reviewing, and editing. MJ: material preparation, conceptualization, and data collection. FF: analysis, interpretation, first draft of the manuscript, conceptualization, and investigation. All authors contributed to the study conception, design, investigation, reviewed and commented on previous versions of the manuscript, and read and approved the final manuscript.

## Conflict of Interest

The authors declare that the research was conducted in the absence of any commercial or financial relationships that could be construed as a potential conflict of interest.

## Publisher's Note

All claims expressed in this article are solely those of the authors and do not necessarily represent those of their affiliated organizations, or those of the publisher, the editors and the reviewers. Any product that may be evaluated in this article, or claim that may be made by its manufacturer, is not guaranteed or endorsed by the publisher.
